# Acute Mesenteric Ischemia after Cardiac Surgery: An Analysis of 52 Patients

**DOI:** 10.1155/2013/631534

**Published:** 2013-10-27

**Authors:** Cuneyt Eris, Senol Yavuz, Serhat Yalcinkaya, Arif Gucu, Faruk Toktas, Gunduz Yumun, Burak Erdolu, Ahmet Ozyazıcıoglu

**Affiliations:** ^1^Departments of Cardiovascular Surgery, Bursa Yuksek Ihtisas Education and Research Hospital, 16330 Bursa, Turkey; ^2^Departments of Thoracic Surgery, Bursa Yuksek Ihtisas Education and Research Hospital, 16330 Bursa, Turkey

## Abstract

*Objective*. Acute mesenteric ischemia (AMI) is a rare but serious complication after cardiac surgery. The aim of this retrospective study was to evaluate the incidence, outcome, and perioperative risk factors of AMI in the patients undergoing elective cardiac surgery. *Methods*. From January 2005 to May 2013, all patients who underwent cardiac surgery were screened for participation, and patients with registered gastrointestinal complications were retrospectively reviewed. Univariate analyses were performed. *Results*. The study included 6013 patients, of which 52 (0.86%) patients suffered from AMI, 35 (67%) of whom died. The control group (150 patients) was randomly chosen from among cases undergoing cardiopulmonary bypass (CPB). Preoperative parameters including age (*P* = 0.03), renal insufficiency (*P* = 0.004), peripheral vascular disease (*P* = 0.04), preoperative inotropic support (*P* < 0.001), poor left ventricular ejection fraction (*P* = 0.002), cardiogenic shock (*P* = 0.003), and preoperative intra-aortic balloon pump (IABP) support (*P* = 0.05) revealed significantly higher levels in the AMI group. Among intra- and postoperative parameters, CPB time (*P* < 0.001), dialysis (*P* = 0.04), inotropic support (*P* = 0.007), prolonged ventilator time (*P* < 0.001), and IABP support (*P* = 0.007) appeared significantly higher in the AMI group than the control group. *Conclusions*. Prompt diagnosis and early treatment should be initiated as early as possible in any patient suspected of AMI, leading to dramatic reduction in the mortality rate.

## 1. Introduction

Abdominal complications after CPB for cardiac surgery are seen with an incidence of 0.4–2.9%, and acute mesenteric ischemia (AMI) represents 10%–67% of these complications [1–4]. Although infrequent, it is one of the serious complications of cardiac surgery characterized by extremely high mortality rates (40% to 94%). This is attributed to delayed diagnosis and ineffective treatment choices [5–7].

There is a risk of delayed diagnosis and treatment because such patients are often intubated and sedated, and, therefore, they are unable to alert the clinician about their symptoms and the intestinal ischemia may not become clinically evident until hours or days.

There are four common causes of AMI: acute embolism to the superior mesenteric artery, acute thrombosis of an atherosclerotic plaque with previous partial occlusion, splanchnic vasoconstriction leading to low flow and regional ischemia that is called nonocclusive mesenteric ischemia (NOMI), and mesenteric venous thrombosis [8]. 

Intestinal ischemia after cardiac surgery most often is due to a NOMI, and it is related to a reduction in the splanchnic blood flow, which can be due to low cardiac output, and it may also be aggravated by inotropic support, such as vasopressors, and by preexisting atherosclerosis [9, 10]. The ischemia due to arterial emboli, arterial thrombosis, or venous thromboembolism is less commonly seen after CPB [11].

There are many demographic and surgical variables seen as risk factors for intestinal ischemia after CPB, such as arterial hypotension, postoperative heart failure, renal insufficiency, age >70 years, hypovolemia, cardiopulmonary bypass time >150 min, New York Heart Association (NYHA) class III-IV, active smoker, sepsis, IABP support, use of vasopressors, and preexisting atherosclerotic lesions [12, 13].

The clinical presentation of AMI can be misleading, while the laboratory and radiological diagnostic tests often produce inadequate results. Plain abdominal radiographs are of little help in the diagnosis of mesenteric ischemia. Multidetector computed tomography angiography (CTA) represents a fast and accurate investigation tool for the diagnosis of AMI. Angiography is the gold standard diagnostic test in acute mesenteric artery occlusion, providing both anatomical visualization of the vessels and therapeutic options [14, 15].

When a diagnosis of AMI is made, treatment should be initiated without delay. Survival is directly related to the degree of bowel ischemia and the extent of bowel resection, but the diagnosis time of AMI is much more important. 

The aim of this study was to investigate the incidence of AMI after cardiac surgery and to identify risk factors, therapeutic options for the disease, and patient outcome.

## 2. Methods

We performed a retrospective analysis on 6013 consecutive patients undergoing CPB for open heart surgery, between January 1, 2005, and May 31, 2013. We excluded patients who had coronary artery bypass grafting (CABG) without extracorporeal circulation, patients younger than 20 years old, and patients operated on for type I aortic dissection. A total of 52 (0.86%) patients had intervention due to mesenteric ischemia, based on clinical and/or laboratory and/or radiological findings. In order to assess probable relationships between such findings and AMI, a control group consisting of 150 patients was randomly chosen from among the rest of the CPB patients.

CABG (*n* = 4338) was the most common procedure, followed by various heart valve procedures (*n* = 1290), CABG and heart valve procedures combined (*n* = 235), and various procedures, for example, postinfarction septal rupture, ascending aortic aneurysm, adult ventricular septal defect (VSD), or atrial septal defect (ASD) (*n* = 150).

The patient record form contained 20 preoperative and 11 intra- and postoperative data defined according to EuroSCORE and previously described risk factors for development of gastrointestinal (GI) complications in the literature [16].

Demographic and clinical characteristics of the patients and preoperative and postoperative data between patients with and without intestinal ischemia are compared in Tables 1 and 2.

Prolonged ventilator time was defined as the use of a ventilator for more than 24 h after cardiac surgery. The need for inotropic support was registered if the patient required one or more inotropic drugs, for example, norepinephrine, dobutamine, and dopamine, for more than 24 h. Postoperative renal failure was defined as a serum creatinine level of above 2 mg/dL. Intestinal ischemia was defined as ischemia diagnosed at endoscopy, abdominal surgery, or mesenteric angiography. Cardiac infarction was defined as elevation of cardiac enzymes or electrocardiographic changes after the cardiac operation.

Mesenteric angiography was performed when the nonspecific clinical signs of AMI (oliguria, unexplained metabolic acidosis with elevation of lactate levels, high anion gap, decreased oxygenation, or hypotension) with or without peritoneal signs were present. The method of mesenteric angiography included lateral aortography and catheterization of superior mesenteric artery (SMA).

Treatment options were determined according to the type of mesenteric ischemia in most of the situations. But if any peritoneal signs at any time during evaluation appeared in any patient, urgent exploratory laparoscopy or laparotomy without delay was the only choice of our treatment. If any uncertainties about bowel viability were assessed, it is safer to perform a laparotomy to check for bowel viability in patients with signs of peritonitis and rebound tenderness. Standard surgical therapy for AMI involved resection of irreparably damaged bowel and reestablishment of mesenteric blood flow through embolectomy, or thrombectomy, endarterectomy or with antegrade percutaneous stenting.

For patients who had findings of trombosis on angiography, without peritoneal signs, selective mesenteric angiography and local thrombolytic therapy (LTT) were initiated immediately as another treatment option. LTT was initiated with recombinant plasminogen activator (rt-PA, ActilyseW, Boehringer Ingelheim GmbH) of 5 mg bolus, followed by 1 mg/h maintenance. After 24 h of treatment, another angiography was performed and the catheter was withdrawn.

The primary treatment for NOMI was medical, with extensive critical care support and prompt arteriography. When we determined NOMI at the angiography, we catheterized the SMA and started intra-arterial infusion of papaverine at 30–60 mg/hour via the angiography catheter. Also volume resuscitation, broad-spectrum antibiotics, and anticoagulants were used for adjuvant therapy. Initial anticoagulation with heparin was the treatment of choice in patients without peritoneal signs. When these patients developed peritoneal signs during admission, surgical exploration was undertaken to assess bowel viability. If necrotic bowel segments were found, intestinal resection with anastomosis or enterostomies was performed, and a second look procedure was planned after 24 h.

The study protocol was approved by the institutional ethics committee. No image of an individual patient is accompanied. Written informed consent was not obtained from the patients for publication of this report or any accompanying images, since we report a large population and not an individual patient. No image of an individual patient is accompanied.

### 2.1. Statistical Analysis

Statistical analyses were performed using MedCalc version 12.7.1 (Licensed to MedcalcTurkey 041021151133). Absolute number and percentages of categorical variables and medians, averages, and standard deviations of continuous variables were determined. Mann-Whitney *U*-test and Wilcoxon test were used where appropriate. A probability level of less than 0.05 was considered significant.

## 3. Results

When we investigated the preoperative datas, fifty-two patients, including 33 (65%) men and 19 (35%) women, were diagnosed as having intestinal ischemia during the study period. 100 (67%) patients were men and 50 (33%) were women in the control group. Body mass index (BMI) was 26.1 ± 4.5 kg/m² in the control group and 25.3 ± 2.7 kg/m² in the AMI group. Preoperative clinical characteristic data between patients with and without intestinal ischemia are compared in Table 1. No significant difference was obtained for gender, BMI, hypertension, hyperlipidemia, chronic obstructive pulmonary disease, and atrial fibrillation between the groups. Age was significantly older in the AMI group than that in the control group (66.2 ± 7.5 years versus 62.1 ± 10.8 years) (*P* = 0.03). The rate of smokers (65% versus 28%) was found significantly higher (*P* = 0.02) in the AMI group. The number of patients who needed inotropic support was 23 (45%) versus 5 (3%) (*P* < 0.001), and the rate of patients with LVEF <30 (34% versus 9%) (*P* = 0.002) was significantly higher in the AMI group. Patients in the AMI group also had significantly higher incidence of NYHA 4 (*P* = 0.006), PVD (*P* = 0.04), renal insufficiency (*P* = 0.004), and cardiogenic shock (*P* = 0.003) than the control group (see Table 1).

Thirty-five (67%) of the patients died within 30 days due to the complications. Physical examination revealed positive peritonitis signs in 35 (67%) of the patients, while there were no peritonitis signs in 17 (33%) of the patients.

When we investigated the intra- and postoperative results, CPB time was 121.7 ± 26.9 minutes in the AMI group and 90.3 ± 16.5 minutes in the control group, significantly longer (*P* < 0.001) in the AMI group. Patients in the AMI group also had significantly higher duration of ventilator times (*P* < 0.001) than those in and the control group. Dialysis due to renal insufficiency (*P* = 0.04), inotropic support (*P* = 0.007), and IABP support (*P* = 0.007) appeared significantly higher in the AMI group than the control group (Table 2).

**Figure 1:**
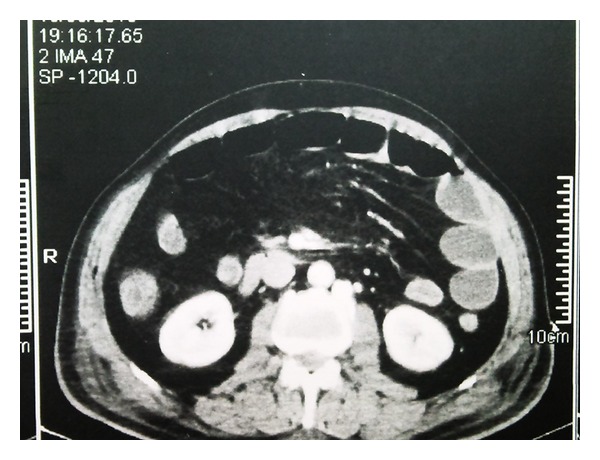
Thickened bowel walls and dilated fluid-filled bowel loops of an AMI patient on an abdominal CT image. AMI: acute mesenteric ischemia; CT: computerized tomography.

**Figure 2:**
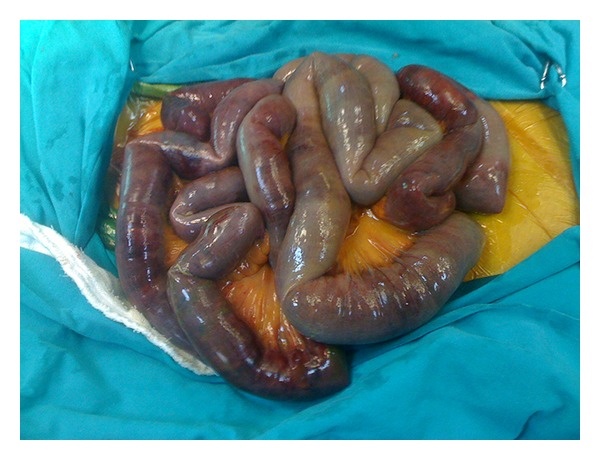
Intraoperative image of a gangrenous and necrotic bowel.

**Figure 3:**
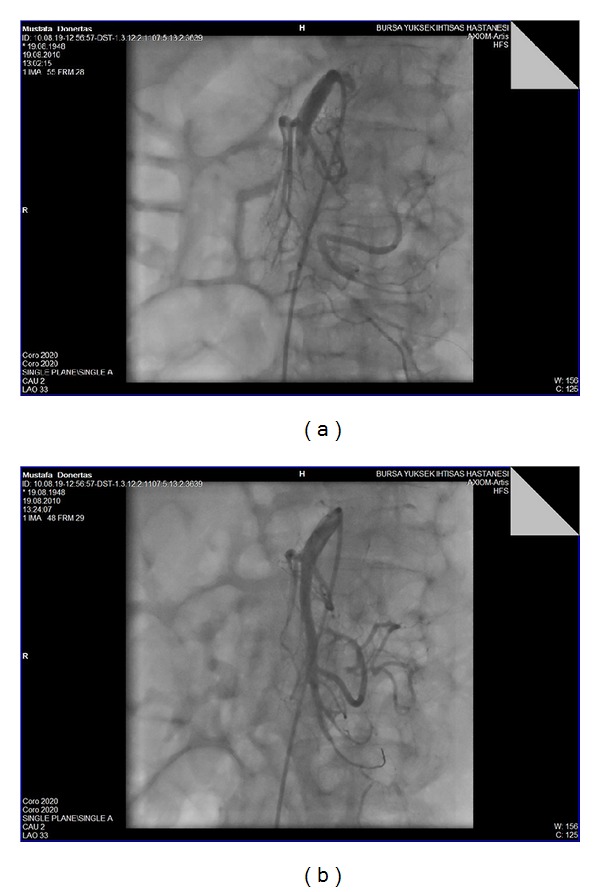
Angiographic appearance of the superior mesenteric artery. (a) Angiographic image of a thrombotic SMA. (b) Angiographic image of the patient after implantation of the stents. SMA: superior mesenteric artery.

Abdominal X-ray and abdominal ultrasonography were performed in all patients. All showed signs of paralysis with dilation of the intestines. CT was performed in 21 (40%) cases (Figure 1). Mesenteric angiography was performed in 35 (67%) patients, and CTA was performed in 7 (13%) out of 52 patients. Ten (19%) patients directly underwent necrotic bowel resection (Figure 2). Five (9%) patients underwent thromboembolectomy. LTT was performed in 5 (9%) patients. In 3 (6%) patients after SMA, stenting LTT was initiated for 24 hours (Figure 3(a)). After 24 hours, a control angiography was performed and revealed recanalization of SMA (Figure 3(b)). There were no signs of peritoneal irritation in these patients; therefore, second-look laparoscopy was not planned.

NOMI was detected in 25 (48%) patients, and 16 (31%) of them had no peritoneal signs in the beginning. Peritoneal signs became positive during the medical treatment in 4 of 16 NOMI patients. The NOMI patients presented with peritoneal signs underwent laparoscopy and subsequently laparotomy when positive findings for possible bowel necrosis were revealed during laparoscopy.

Patients who had low-flow state without bowel necrosis during the evaluation did not undergo bowel resection. In these patients, a second-look laparotomy was associated with partial bowel resection if findings for possible bowel necrosis were revealed. The applied interventions, the number of patients, and mortality are presented in Table 3.

Among preoperative parameters, age (*P* = 0.03), renal insufficiency (*P* = 0.004), preoperative inotropic treatment >24 h (*P* < 0.001), smoking (*P* = 0.02), emergency cardiac surgery (*P* = 0.01), PVD (*P* = 0.04), LVEF <30 (*P* = 0.002), cardiogenic shock (*P* = 0.003), NYHA (*P* = 0.006), and IABP support (*P* = 0.05) were found to be significantly higher in the AMI group (Table 1).

When we investigate the intra- and postoperative values, CPB time (*P* < 0.001), renal insufficiency (*P* < 0.001), the need for dialysis (*P* = 0.04), IABP support (*P* = 0.007), prolonged ventilator time (*P* < 0.001), inotropic treatment >24 h (*P* = 0.007), and cardiac infarction (*P* = 0.03) were found significantly higher in the AMI group (see Table 2).

## 4. Discussion

In the present study we investigated retrospectively the patients who had developed AMI following CPB.

We found the incidence of 0.86% for AMI in our patient population, and in this group the mortality rate was 67%. We observed the similar rates when compared with the studies previously presented by the other authors [13, 15]. Risk factors were identified that can aid in the diagnosis. To reduce the delay in diagnosis and allow effective use of all therapeutic options, a high index of suspicion for intestinal ischemia after cardiac surgery is warranted, in order to reduce mortality. In particular, in a patient with a septic condition early after surgery, this diagnosis must always be considered.

The clinical signs and biochemical or hematological markers of AMI are nonspecific. Even at the time when ischemia is confirmed at laparotomy, elevation of serum lactate, amylase, creatine kinase, and C-reactive protein (CRP), as well as leucocytes, may be absent. At present, no laboratory test is available for accurately establishing or eliminating the diagnosis [17].

Direct abdominal radiographs are of little help in the diagnosis of mesenteric ischemia. The presence of dilated loops is nonspecific, and thickened bowel loops, “ground-glass” appearance suggesting ascites, or “thumbprinting” caused by submucosal edema or hemorrhage is seen in less than 40% of patients [18]. Doppler sonography is useful in diagnosing chronic mesenteric arterial occlusive disease but has limited role in AMI. By the help of color doppler sonography, the flow velocities and resistance index in the splanchnic arteries and end-organ vascularity can be evaluated as well [19]. We performed color doppler sonography on every patient who had suspicion of AMI. Also abdominal CT has poor sensitivity and specificity in the diagnosis of most types of AMI. With the help of CT, nonspecific findings like thickened bowel walls, intramural hematoma, dilated fluid-filled bowel loops, engorgement of mesenteric vessels, pneumatosis, mesenteric or portal venous gas, infarction of other viscera, and arterial or venous thrombus can be seen [20]. We performed abdominal CT in 21 (40%) patients who had nonspecific clinical signs of AMI.

Multidetector CT angiography represents a fast and accurate investigation tool for the diagnosis of AMI [21]. In most cases, it can be used as the sole diagnostic procedure. This method provides direct visualization of the mesenteric vasculature, intestines, and mesentery, but when compared with conventional angiography, the disadvantage is the lack of therapeutic option. In our patients, we preferred to use CTA as a first diagnostic step in 7 (13%) patients.

Angiography is the gold standard diagnostic test in acute mesenteric artery occlusion by providing both anatomical visualization of the vessels and therapeutic options (possibility of intravascular administration of vasodilators and thrombolytics) [22].

Mesenteric angiography can usually differentiate embolic from thrombotic arterial occlusions. NOMI characteristically shows narrowing and multiple irregularities of the major SMA tributaries, the “string of sausages” sign at the angiography. Venous occlusion and NOMI may show contrast material refluxing back into the aorta on selective SMA angiography.

Klotz et al. demonstrated that the indication for selective mesenteric angiography is established if at least one of four possible indications for mesenteric ischemia is present: (i) no defecation for more than 3 days after surgery, despite maximal laxative treatment, (ii) severe abdominal bloating with a considerably distended belly, (iii) clinical and radiologic signs of paralytic ileus, or (iv) borderline or elevated serum lactate [15].

We used the same indications as Klotz et al. in our daily practice for selective mesenteric angiography.

Patients with intestinal ischemia after cardiac surgery often have vague and nonspecific symptoms. Abdominal pain is the primary symptom and other symptoms, are present inconstantly including nausea, vomiting, and diarrhea [23]. Physical examination is unremarkable unless peritonitis has developed. During the late stages, abdominal distension and guarding, as well as systemic complications, may be encountered. The most common laboratory abnormalities are unexplained metabolic acidosis with elevation of lactate level and high anion gap, hemoconcentration, and leukocytosis [24].

When a diagnosis of AMI is made, treatment should be initiated without delay. After volume resuscitation, broad-spectrum antibiotics, vasodilators, and intravenous heparin at therapeutic doses should be initiated as early as possible [25]. Although surgical revascularization is the standard procedure, embolectomy, thrombectomy, and endarterectomy, as well as endovascular techniques such as balloon angioplasty, antegrade percutaneous stenting, thrombolysis, and percutaneous thrombus extraction, can all be used to restore luminal visceral blood flow with good short-term outcome [26–28].

If the patient develops signs of bowel infarction such as peritonitis, worsening sepsis, or metabolic acidosis during treatment, laparotomy is indicated. In our study group, there were 10 patients who had high index of suspicion of AMI, directly taken to the operation because of the acute onset of peritoneal signs positivity.

Safioleas et al. reported that in patients with arterial occlusive AMI, when sufficient bowel is potentially viable, revascularization prior to resection of the infarcted bowel may improve the survival [29]. Urgent exploratory laparotomy or laparoscopy is the key to successful management. However, early laparotomies do not necessarily mean survival in cases of extensive ischemia [9].

In NOMI, diffuse vasospasm of the mesenteric and other visceral arteries occurs as a result of a sustained hypoperfusion state [30]. No vascular occlusion is usually demonstrated because pulsatile blood flow is present in larger arteries.

We had 25 (48%) angiographically proven NOMI cases, which was similar to values previously presented after the CPB [9, 10]. Angiographically proven NOMI can be treated with intra-arterial infusion of tolazoline, papaverine, or prostaglandin E2, after the selective intra-arterial catheterization of the SMA [31]. We used papaverine infusion for 24–48 hours at our clinic practice. Even if bowel resection is required, we continued papaverine infusion postoperatively to guard against persistence of vasospasm which may lead to further bowel ischemia and infarction.

Previous studies have identified age >70 years, severe heart failure, cardiogenic shock, and chronic renal insufficiency as important preoperative risk factors for the development of mesenteric ischemia [13, 24]. In the present study, on investigating our values, we found that most of the results were in compliance with previous studies. In a recent study, Nilsson et al. found that advanced age was not a risk factor for AMI, but we found older age significantly higher in our AMI group than the control group [32]. The preoperative inotropic support was found to be more common in patients with AMI but was not pointed out as a risk factor at previous studies.

The other remarkable postoperative factors described in the literature are hypovolemic shock, cardiogenic shock, administration of *α*-adrenergic drugs, underlying atherosclerotic disease, and the use of an IABP [31, 32].

Vasopressor treatment is necessary in cardiac patients before, during, and after operations involving CPB [33]. Treatment with *α*-adrenergic catecholamines like norepinephrine and high doses of dopamine may lead to an increased incidence of NOMI after CPB [34]. These changes may be due to constriction of the intestinal mucosal arterioles as a consequence of *α*-adrenoceptor stimulation. In addition, norepinephrine simulates in a dose-dependent manner *β*-receptors, which has been shown to increase intestinal oxygen consumption [35]. In the present study, nearly half of the patients before the operation and 85% of the patients in postoperative period were treated with inotropes at the AMI group. These rates were significantly higher than the control group.

IABP implantation after cardiac surgery is a commonly used form of circulatory support for patients with postoperative low cardiac output syndrome. Despite improving coronary perfusion and reducing left ventricular afterload, IABP use is known as a risk factor for the development of lethal mesenteric ischemia [36]. The malposition of the IABP balloon is probably the factor initiating in compromised visceral blood flow. In a recent study, the authors found a malposition of the IABP balloon with consecutive visceral artery compromises in up to 97% of the patients, and laparotomy for mesenteric ischemia was required in 23.8% [37]. Therefore after insertion of the IABP, the position was immediately confirmed by chest X-ray film. We determined a malposition of the IABP balloon during angiography only in 2 patients (4%), which was then corrected. Rather than malposition, the number of patients whose IABP was inserted was significantly higher than that of the control group in our AMI group. According to our results, even if IABP is correctly positioned, IABP balloon may aggravate a state of intestinal hypoperfusion.

The mortality rate among patients with acute mesenteric ischemia remains high. Schoots et al. in a recent study reported an overall mortality rate of almost 95% for nonsurgically treated patients compared to approximately 57% for surgically treated patients [6]. In our study, thirty five (67%) of the patients died within 30 days due to the complications. Unlike their results, our mortality rate was 64% (9/14) for medically treated group and 68% (26/38) for both surgically and medically treated group.

## 5. Conclusions

Acute mesenteric ischemia is a challenging clinical problem with various causes, which often results in delayed diagnosis and treatment. We consider that, regardless of the results of the diagnostic tests, immediate mesenteric angiography and aggressive appropriate early treatment are more successful than conservative management of these patients. Therefore, these procedures should be initiated as early as possible in any patient suspected of having mesenteric ischemia, leading to dramatic reduction in the mortality rate.

Poor preoperative cardiac condition, renal insufficiency, peripheral vascular disease, urgent cardiopulmonary bypass, and IABP support were to be the independent prognostic factors for the development of this complication.

## Figures and Tables

**Table 1:** Preoperative values for intestinal ischemia after cardiac surgery.

Variable	Control group *n* = 150	Patients with intestinal ischemia *n* = 52	*P* value
Age (years)*	62.1 ± 10.8	66.2 ± 7.5	0.03
Gender, male/female	100/50 (67/33)	33/19 (65/35)	0.87
BMI (kg/m²)*	26.1 ± 4.5	25.3 ± 2.7	0.25
Hypertension	42 (28)	17 (32)	0.85
Creatinine >2 (mg/dL)	5 (3)	14 (28)	0.004
Dialysis	1 (1)	0	1
Inotropic treatment >24 h	5 (3)	23 (45)	<0.001
Smoking	42 (28)	34 (65)	0.02
Emergency cardiac surgery	14 (9)	17 (33)	0.01
Diabetes mellitus	35 (23)	14 (26)	0.70
Chronic obstructive pulmonary disease	14 (9)	7 (13)	0.46
Stroke	6 (4)	3 (6)	1
Peripheral vascular disease	15 (10)	16 (30)	0.04
Left ventricular ejection fraction <30%	14 (9)	18 (34)	0.002
Cardiogenic shock	3 (2)	15 (29)	0.003
Unstable angina	17 (11)	7 (13)	0.46
IABP	6 (4)	8 (16)	0.05
Atrial fibrillation	14 (9)	8 (16)	0.83
NYHA 4	14 (9)	21 (41)	0.006
Hyperlipidemia	20 (13)	6 (12)	0.83

BMI: body mass index, IABP: intra-aortic balloon pump, NYHA: New York Heart Association.

*n* is the number of nonmissing values; values in parentheses are percentages. *Data are presented as the mean ± SD where indicated.

**Table 2:** Perioperative values for intestinal ischemia after cardiac surgery.

Variable	Control group (*n* = 150)	Patients with intestinal ischemia (*n* = 52)	*P* value
CPB time (minutes)*	90.3 ± 16.5	121.7 ± 26.9	<0.001
Creatinine >2 (mg/dL)	8 (5)	32 (62)	<0.001
Dialysis	3 (2)	10 (19)	0.04
IABP	9 (6)	12 (23)	0.007
Reoperation due to bleeding	5 (3)	3 (6)	0.81
Prolonged ventilator time >24 h	14 (9)	36 (69)	<0.001
Inotropic treatment >24 h	18 (12)	44 (85)	0.007
Atrial fibrillation	38 (25)	17 (33)	0.26
Arrhythmia	48 (32)	19 (36)	0.73
MI	5 (3)	9 (18)	0.03
Cerebrovascular event	5 (3)	2 (4)	1

*n* is the number of nonmissing values; values in parentheses are percentages, except the mean ± SD *CPB: cardiopulmonary bypass, IABP: intra-aortic balloon pump, and MI: myocardial infarction.

**Table 3:** Abdominal and vascular interventions on patients with intestinal ischemia.

Abdominal and vascular interventions	Patients (*n* = 52)	Mortality
Necrotic bowel resection	*n* = 14	*n* = 13
Thromboembolectomy	*n* = 2	*n* = 1
Thromboembolectomy + bowel resection	*n* = 3	*n* = 2
LTT + stent implantation	*n* = 3	*n* = 1
LTT + bowel resection	*n* = 3	*n* = 1
LTT	*n* = 2	*n* = 1
NOMI (medical treatment)	*n* = 12	*n* = 8
NOMI (medical treatment + bowel resection)	*n* = 13	*n* = 8

*n*: number.

LTT: local thrombolytic therapy; NOMI: nonocclusive mesenteric ischemia.

## Abbreviations

BMI: Body mass index
CABG: Coronary artery bypass grafting
CPB: Cardiopulmonary bypass
LVEF: Left ventricular ejection fraction
AMI: Acute mesenteric ischemia
CT: Computed tomography
CVI: Cerebrovascular insult
GI: Gastrointestinal
LTT: Local thrombolytic therapy
IABP: Intra-aortic balloon pump
NOMI: Nonocclusive mesenteric ischemia
NYHA: New York Heart Association
SMA: Superior mesenteric artery
CTA: Computed tomography angiography
PVD: Peripheral vascular disease.

## References

[1] Mangi AA, Christison-Lagay ER, Torchiana DF (2005). Gastrointestinal complications in patients undergoing heart operation: an analysis of 8709 consecutive cardiac surgical patients. *Annals of Surgery*.

[2] Edwards M, Sidebotham D, Smith M, Leemput JV, Anderson B (2005). Diagnosis and outcome from suspected mesenteric ischaemia following cardiac surgery. *Anaesthesia and Intensive Care*.

[3] Bolcal C, Iyem H, Sargin M (2005). Gastrointestinal complications after cardiopulmonary bypass: sixteen years of experience. *Canadian Journal of Gastroenterology*.

[4] Filsoufi F, Rahmanian PB, Castillo JG, Scurlock C, Legnani PE, Adams DH (2007). Predictors and outcome of gastrointestinal complications in patients undergoing cardiac surgery. *Annals of Surgery*.

[5] Acosta-Merida MA, Marchena-Gomez J, Hemmersbach-Miller M, Roque-Castellano C, Hernandez-Romero JM (2006). Identification of risk factors for perioperative mortality in acute mesenteric ischemia. *World Journal of Surgery*.

[6] Schoots IG, Koffeman GI, Legemate DA, Levi M, van Gulik TM (2004). Systematic review of survival after acute mesenteric ischaemia according to disease aetiology. *British Journal of Surgery*.

[7] Merle C, Lepouse C, de Garine A (2004). Surgery for mesenteric infarction: prognostic factors associated with early death within 72 hours. *Journal of Cardiothoracic and Vascular Anesthesia*.

[8] Park WM, Gloviczki P, Cherry KJ (2002). Contemporary management of acute mesenteric ischemia: factors associated with survival. *Journal of Vascular Surgery*.

[9] Hasan S, Ratnatunga C, Lewis CT, Pillai R (2004). Gut ischaemia following cardiac surgery. *Interactive Cardiovascular and Thoracic Surgery*.

[10] Kazui T, Yamasaki M, Abe K, Watanabe S, Kawazoe K (2012). Non-obstructive mesenteric ischemia: a potentially lethal complication after cardiovascular surgery: report of two cases. *Annals of Thoracic and Cardiovascular Surgery*.

[11] Albes JM, Schistek R, Baier R, Unger F (1991). Intestinal ischemia associated with cardio-pulmonary-bypass surgery: a life threatening complication. *Journal of Cardiovascular Surgery*.

[12] Pang PY, Sin YK, Lim CH, Su JW, Chua YL (2012). Outcome and survival analysis of intestinal ischaemia following cardiac surgery. *Interactive Cardiovascular and Thoracic Surgery*.

[13] Chaudhuri N, James J, Sheikh A, Grayson AD, Fabri BM (2006). Intestinal ischaemia following cardiac surgery: a multivariate risk model. *European Journal of Cardio-Thoracic Surgery*.

[14] Garofalo M, Borioni R, Nardi P (2002). Early diagnosis of acute mesenteric ischemia after cardiopulmonary bypass. *Journal of Cardiovascular Surgery*.

[15] Klotz S, Vestring T, Rötker J, Schmidt C, Scheld HH, Schmid C (2001). Diagnosis and treatment of nonocclusive mesenteric ischemia after open heart surgery. *The Annals of Thoracic Surgery*.

[16] Andersson B, Andersson R, Brandt J, Höglund P, Algotsson L, Nilsson J (2010). Gastrointestinal complications after cardiac surgery—improved risk stratification using a new scoring model. *Interactive Cardiovascular and Thoracic Surgery*.

[17] Woo K, Major K, Kohanzadeh S, Allins AD (2007). Laparotomy for visceral ischemia and gangrene. *American Surgeon*.

[18] Chien-Hua L, Jyh-Cherng Y, Huan-Fa H, Hurng-Sheng W, Shih-Yi C, Chu-Hsin C (2007). Pneumatosis intestinalis and hepatic-portal-mesenteric venous gas in intestinal ischemia. *Revista Espanola de Enfermedades Digestivas*.

[19] Hamada T, Yamauchi M, Tanaka M, Hashimoto Y, Nakai K, Suenaga K (2007). Prospective evaluation of contrast-enhanced ultrasonography with advanced dynamic flow for the diagnosis of intestinal ischaemia. *The British Journal of Radiology*.

[20] Fock CM, Kullnig P, Ranner G, Beaufort-Spontin F, Schmidt F (1994). Mesenteric arterial embolism—the value of emergency CT in diagnostic procedure. *European Journal of Radiology*.

[21] Ofer A (2009). Multidetector CT angiography in the evaluation of acute mesenteric ischemia. *European Radiology*.

[22] Acosta S, Sonesson B, Resch T (2009). Endovascular therapeutic approaches for acute superior mesenteric artery occlusion. *CardioVascular and Interventional Radiology*.

[23] Kassahun WT, Schulz T, Richter O, Hauss J (2008). Unchanged high mortality rates from acute occlusive intestinal ischemia: six year review. *Langenbeck’s Archives of Surgery*.

[24] Ghosh S, Roberts N, Firmin RK, Jameson J, Spyt TJ (2002). Risk factors for intestinal ischaemia in cardiac surgical patients. *European Journal of Cardio-Thoracic Surgery*.

[25] Kozuch PL, Brandt LJ (2005). Review article: diagnosis and management of mesenteric ischaemia with an emphasis on pharmacotherapy. *Alimentary Pharmacology and Therapeutics*.

[26] Gartenschlaeger S, Bender S, Maeurer J, Schroeder RJ (2008). Successful percutaneous transluminal angioplasty and stenting in acute mesenteric ischemia. *CardioVascular and Interventional Radiology*.

[27] Moyes LH, McCarter DHA, Vass DG, Orr DJ (2008). Intraoperative retrograde mesenteric angioplasty for acute occlusive mesenteric ischaemia: a case series. *European Journal of Vascular and Endovascular Surgery*.

[28] Karwowski J, Arko F (2004). Surgical management of mesenteric ischemia. *Techniques in Vascular and Interventional Radiology*.

[29] Safioleas MC, Moulakakis KG, Papavassiliou VG, Kontzoghu K, Kostakis A (2006). Acute mesenteric ischaemia, a highly lethal disease with a devastating outcome. *Vasa*.

[30] Imanaka K, Kyo S, Abe K (2006). Severe hepatic artery spasm and nonocclusive mesenteric ischemia after cardiac surgery. *The Annals of Thoracic Surgery*.

[31] Huwer H, Winning J, Straub U, Isringhaus H, Kalweit G (2004). Clinically diagnosed nonocclusive mesenteric ischemia after cardiopulmonary bypass: retrospective study. *Vascular*.

[32] Nilsson J, Hansson E, Andersson B (2013). Intestinal ischemia after cardiac surgery: analysis of a large registry. *Journal of Cardiothoracic Surgery*.

[33] Khan TA, Bianchi C, Ruel M, Feng J, Sellke FW (2007). Differential effects on the mesenteric microcirculatory response to vasopressin and phenylephrine after cardiopulmonary bypass. *Journal of Thoracic and Cardiovascular Surgery*.

[34] O’Dwyer C, Woodson LC, Conroy BP (1997). Regional perfusion abnormalities with phenylephrine during normothermic bypass. *The Annals of Thoracic Surgery*.

[35] Salak N, Pajk W, Knotzer H (2001). Effects of epinephrine on intestinal oxygen supply and mucosal tissue oxygen tension in pigs. *Critical Care Medicine*.

[36] Venkateswaran RV, Charman SC, Goddard M, Large SR (2002). Lethal mesenteric ischaemia after cardiopulmonary bypass: a common complication?. *European Journal of Cardio-Thoracic Surgery*.

[37] Rastan AJ, Tillmann E, Subramanian S (2010). Visceral arterial compromise during intra-aortic balloon counterpulsation therapy. *Circulation*.

